# Tripartite motif-containing 28 (TRIM28) expression and cordycepin inhibition in progression, prognosis, and therapeutics of patients with breast invasive carcinoma

**DOI:** 10.7150/jca.95876

**Published:** 2024-06-11

**Authors:** Dabing Li, Jingliang Cheng, Wenqian Zhang, Lianmei Zhang, Mazaher Maghsoudloo, Jiewen Fu, Xiaoyan Liu, Xiuli Xiao, Chunli Wei, Junjiang Fu

**Affiliations:** 1 Key Laboratory of Epigenetics and Oncology, the Research Center for Preclinical Medicine, Southwest Medical University, Luzhou 646000, Sichuan Province, China.; 2 School of Basic Medical Sciences, Southwest Medical University, Luzhou 646000, Sichuan Province, China.; 3 Department of Pathology, The Affiliated Huai'an No. 1 People's Hospital of Nanjing Medical University, Huai'an 223300, Jiangsu Province, China.; 4 Department of Pathology, the Affiliated Hospital, Southwest Medical University, Luzhou 646000, Sichuan Province, China.

**Keywords:** The *TRIM28* gene, Expression, Breast invasive carcinoma (BRCA), Metastasis, Prognostics

## Abstract

Breast cancer (BC) is the most common tumor in women worldwide. TRIM28 (RNF96) plays pleiotropic biological functions, such as silencing target genes, facilitating DNA repair, stimulating cellular proliferation and differentiation, and contributing to cancer progression. TRIM28 plays an increasingly crucial role in cancer, but its impact on BC, including breast invasive carcinoma, remains poorly understood. In the current study, analyses of online databases, quantitative real-time quantitative PCR, immunohistochemistry, and western blotting were performed on patients with breast invasive carcinoma (BRCA). Cordycepin (CD) was used to monitor BC progression and TRIM28 expression *in vivo*. As a result, we observed that TRIM28 is highly expressed in breast invasive carcinoma tissues compared with the corresponding normal tissues and is correlated with metastatic / invasive progression. High expression of TRIM28 might serve as a prognostic marker for long-term survival in triple-negative BC, advanced BC, or breast invasive carcinoma. Although TRIM28 methylation in tumor tissues of breast invasive carcinoma is not significantly changed compared to the matched normal tissues, the expressions and methylation of TRIM28 are significantly reversely correlated. TRIM28 expression was inhibited by CD in the mouse model, indicating its role in preventing BC progression. Thus, TRIM28 might be a potentially valuable molecular target for forecasting the progression / prognosis of patients with breast invasive carcinoma. CD, which represses BC growth/metastasis, may be involved partially through suppressing TRIM28 expression.

## Introduction

Malignancy cancer shows the most common patient cause of death worldwide and significantly compromises life quality in all countries. In 2024, it is predicted that 2,001,140 new cancer patients and 611,720 patient deaths will occur in the United States 1, representing a slight increase from 2023 2. Breast cancer (BC) shows the most common tumor in women globally. Applicable data indicates that there were approximately 2.3 million new cases of breast cancer and approximately 685,000 dead patients worldwide in 2020 2-4. In China, approximately 416,371 new female BC patients were reported in 2020, constituting 18.41% of new female cases in women worldwide. By 2040, the burden of BC is predicted to increase to over 3 million new patients and over 1 million dead patients each year. While triple-negative breast cancer (TNBC), a pathological subtype of BC defined by the lack of receptor expression for estrogen (ER) and progesterone (PR) and for epidermal growth factor receptor 2 (HER2), or the lack of amplification for HER2, accounts for 15~ 20% of all types in BC 5. Breast invasive carcinoma (BRCA) and TNBC pose the greatest challenges in BC treatment, exhibiting the highest mortality rates, increased metastasis, a higher likelihood of recurrence, and the worst prognosis. Despite some advancements, numerous challenges persist, including the development of novel molecular targets, diagnostic techniques, and therapeutic strategies aimed at inhibiting BC recurrence and metastasis.

Tripartite motif-containing protein 28 (TRIM28), also known as TIF1β (transcriptional intermediary factor 1β), KAP1 (Krüppel-Associated Box (KRAB) domain-associated protein 1), or RNF96 (ring finger protein 96), is considered a significant mediator of carcinogenesis 6. The TRIM28 contains the N terminus of a ring-finger domain, a coiled-coil region, four conserved domains, namely a B-box type 1 and a B-box type 2, collectively referred to as the TRIM domain or RBCC domain, and a C-terminal PHD/Bromodomain 7, 8. The RBCC or TRIM domain was shown to interact with a KRAB domain, which includes a large number of KRAB-Zinc finger transcription factors. The recently discovered B box domain TRIM28 plays roles in L1 retrotransposition and ORF2p-mediated cDNA synthesis 9.

As a transcriptional co-regulator or repressor, TRIM28 plays pleiotropic biological functions, such as silencing target genes, facilitating DNA repair, stimulating cellular proliferation and differentiation, and contributing to cancer progression 10, 11. In a mouse model of prostate cancer, TRIM28 was found to promote luminal cell plasticity 12, supporting its pro-tumorigenic roles in human prostate cancers 10, 13, 14. By promoting the recruitment in myeloid-derived suppressor cells, TRIM28 enhances anti-PD-1 resistance in non-small cell lung cancer 15. TRIM28 forms a complex with TIAM1 to mediate epigenetic silencing of protocadherins, thereby promoting lung cancer cell migration 16. In ovarian cancer, TRIM28 regulates CBX8-mediated cell proliferation and tumor metastasis by recruiting E2F transcription factor 1 (E2F1) 17. In BC cell lines, HIF-1 recruits both TRIM28 and DNA-dependent protein kinase (DNA-PK), promoting HIF-1 transcriptional activity in response to hypoxia 18.

In regulating gene expression, TRIM28 affects the transcriptional activities at KRAB-ZFP-specific loci, engages in trans-repression, and contributes to epigenetic regulation in chromatin structures 19. TRIM28 serves as an E3 ligase, participating in targeted protein ubiquitination and degradation, such as TP53 20. TRIM28 has been reported to safeguard its family member, TRIM24, from SPOP-moderated degradation, thereby mediating the progression in prostate cancer 13. TRIM28 is found to protect TWIST1 degradation, consequently promoting the progression of BC 19. Moreover, TRIM28 increase was reported to be associated with poor prognosis of gastric cancer 21, hepatocellular carcinoma (HCC) 22, 23, and ovarian cancer 24. Through systematic analysis, TRIM28 was reported to act as an immunological and prognostic predictor for immunotherapy resistance 25.

Although TRIM28 is increasingly being found to play crucial roles in cancers, its impact on breast cancer remains poorly understood, and the expression profiles of TRIM28 in pan-cancers have not been systematically investigated. It is also unknown whether the TRIM28 expression is correlated with metastasis and poor prognosis of patients with breast invasive carcinoma. Cordycepin (CD) is an anti-cancer small molecule derived from a natural product of *Ophiocordyceps sinensis* (Berk.) or *Cordyceps militaris* Link. Our group and others have reported that CD exerted effects on tumor progression, including proliferation, migration, and invasion, both *in vitro* and *in vivo* 26-29. It is unknown whether TRIM28 is regulated by CD treatment in BC growth, metastasis, and invasion in mice. In the current study, through analysis of online databases, RT-qPCR, IHC, and western blotting, we revealed that TRIM28 is highly expressed in BRCA tissues and may be associated with metastatic / invasive progression. TRIM28 high expression might be a prognostic marker for long survival in TNBC or advanced / invasive breast carcinoma but further studies are needed to confirm this. Although TRIM28 methylation in BRCA tumor tissues is not significantly changed compared to corresponding normal tissues, there exists a reverse association between TRIM28 expression and methylation. In a mouse model, CD inhibits TRIM28 expression, suggesting its role in preventing BC progression. Thus, TRIM28 might be a potentially valuable, molecular marker for predicting the progression / prognosis for patients with BRCA.

## Materials and Methods

### Online databases

RNA sequencing data for 33 human cancer types from the Genotype Tissue Expression (GTEx) and TCGA databases were obtained from the UCSC XENA website (https://xenabrowser.net/datapages/). GEPIA2 (gene expression profiling interactive analysis) was used for the analysis of the TRIM28 level (http://gepia2.cancer-pku.cn/) 30, 31. DNMIVD (DNA Methylation Interactive Visualization Database) was used to analyze TRIM28 expression and methylation (http://119.3.41.228/dnmivd/). The TRIM28 protein expression in BC was analyzed by UALCAN 32. TNM plotter (https://tnmplot.com/analysis/) was applied to examine TRIM28 expression differences among tumor, metastatic, and normal tissues 33. The Kaplan-Meier plotter was used to monitor the prognosis of breast cancer patients (https://kmplot.com/analysis/index.php?p=service) 34. The "Survival analysis" module was applied to investigate the relationship between TRIM28 expression and clinical outcomes in different BCs, with the auto-select best cutoff for TRIM28 expression chosen for group splitting. Overall survival (OS), recurrence-free survival (RFS), and distant metastasis-free survival (DMFS) data for patients with BC data (Affy ID:200990_at) were acquired through Kaplan-Meier Plotter (https://kmplot.com/analysis/index.php?p=service). The sequences of *TRIM28* in GenBank (access number: NM_005762) at the National Center for Biotechnology Information (NCBI) were applied to design PCR primers (https://bioinfo.ut.ee/primer3-0.4.0/) 35.

### Real-time quantitative RT-PCR (RT-qPCR) assay

Thirty-three breast invasive ductal carcinoma tissues and their corresponding healthy tissues, obtained from individuals aged between 33 and 72 years old 19, 36, were used in the study. Invasive breast cancer was diagnosed by two independent pathologists; all patients had no other malignancies. Total RNA extraction was carried out using the RNeasy® mini kit (Cat No: 74104, Qiagen). The concentration of total RNA was checked by NanoDrop 2000, and the final concentration was adjusted to 500 ng/μl for synthesis of cDNA (reverse transcriptase/RT-PCR) using products from TOYOBO and BIOBRK companies from China. The synthesized cDNAs were then used as the template for RT-qPCR. The TaqMan probes and primers for TRIM28 were obtained from the Universal Probe Library Center (Roche, Germany) (Table 1). The internal control used was 18S RNA (Table 1). The RT-qPCR assay was performed using the StepOne plus Thermocycler (Life Technologies, USA). Relative amounts of TRIM28 mRNA were gained via normalizing to 18S RNA and expressed as 2^-ΔΔCT^ 19. Each experiment was repeated three times.

### Cordycepin (CD) treatments in mice model

Animal model experiments were conducted in strict compliance with university animal care guidelines and carried out according to committee-approved protocols 26, 37. Approximately 5×10^5^ 4T1 cells in 100μl of FBS-free medium were mixed and subcutaneously injected into the 4^th^ fat pads of 6-week-old BALB/c mice, with each mouse weighing between 20~22 grams. All mice were randomly divided into 3 groups. Different dosages (0, 37.5, 75mg/kg/d) of CD 26, 38 were administered via intraperitoneal injection initially after a 5-day injection and then injected 4 days each week. Tumor volumes were calculated using the following formula: tumor volume (mm^3^) = length (mm) × width (mm)^2^ × π/6. At the endpoint of the experiment of 4 weeks 37, the mice were sacrificed, and tumor tissues were excised. Whole proteins were extracted by the ice-cold 1 x EBC buffer (20 mM Tris-HCl, pH 8.0, 125 mM NaCl, 2 mM EDTA, and 0.5% NP-40) containing a protease inhibitor cocktail. Tumor tissues were also applied for routine hematoxylin-eosin (H&E) staining 37.

### Immunohistochemistry (IHC) assay

The aforementioned human tissues were paraffin-embedded and used for IHC staining analysis to assess the protein level of TRIM28, following the standard immunoperoxidase staining procedure 39. The tissues were embedded in paraffin, cut into 5 µm thick slices, and underwent dewaxing and rehydration. The rehydration process included 100% alcohol I~III for 1 min each, and 95% alcohol for 1 min, 75% alcohol for 1 min, followed by washing twice with ddH^2^O. Antigen retrieval was executed at 100 °C using a 10 mM citric acid buffer. Subsequently, tissue sections were incubated with TRIM28 antibody (1:100 dilutions, cat #: sc-18146, H-300, Santa Cruz Biotechnology, USA) at 4 ºC overnight. The next day the slides were incubated with a secondary antibody at room temperature for 2h after washing with 1×PBS twice. Then, the avidin-biotin-peroxidase complex (ABC) was used to incubate at room temperature for 2h, followed by washing thrice with 1× PBS. After reverse staining with hematoxylin, immunoreactivity was observed using diaminobenzidine (DAB). The slides were then dehydrated and preserved with 75% alcohol for 30s, 100% alcohol for 30s, and xylene for 30s, and covered with glass slides. The score closest to the maximum Yoden index was applied for the optimal cut-off value 39. In each slide, the number of positive tumor cells was counted in 10 random fields under a 10 × 40-fold high-power light microscopy. The detailed criteria for defining the frequency and extent of TRIM28 expression was performed in each entire slide which was evaluated under microscopy. First, a proportion score was assigned, which represented the estimated proportion of positive-staining tumor cells (1, ≤25%; 2, 26% to 50%; 3, 51% to 75%; and 4, >75%). Next, an intensity score was assigned, which represented the average intensity of positive tumor cells (0, none; 1, weak; 2, intermediate; and 3, strong). We multiplied the two scores to obtain a total score, with ≤ 6.3 classified as low expression and > 6.3 as high expression. Slides were scored by pathologists who did not have knowledge of ligand-binding results or patient outcomes.

### Western blotting assay

Protein extraction was performed on twelve fresh breast invasive ductal carcinoma tissues and their corresponding healthy tissues. Invasive breast cancer was diagnosed by two independent pathologists as described above. Whole proteins were lysed using ice-cold 1 x EBC buffer. Approximately 50-60 mg of proteins were separated in an 8% SDS-PAGE with 1 x running buffer at 100 V 37, 40. Subsequently, the protein in each well was transferred to a PVDF membrane and sealed at room temperature with 5% fat-free milk for 2h. The membrane was then incubated with TRIM28 antibody (1:3000 dilution, cat #: sc-33186, H-300, Santa Cruz Biotechnology, USA) in 2% fat-free milk at 4 °C overnight. The β-actin antibody (1:5000 Cell Signaling Technology) was set as an internal control. After being washed thrice with 1×TBST, the secondary antibody was incubated at room temperature for 1 h. The secondary antibodies used to include anti-rabbit antibodies (1:2000) for TRIM28 and anti-mouse antibodies (1:2000) for β-actin (Sigma, USA). Subsequently, the PVDF membranes were then washed thrice with 1 x TBST. An imaging scanner was applied to monitor the signals on each membrane strip. Each experiment was repeated three times.

### Statistical analysis

The statistical analysis was carried out by a t-test (two groups), expressed as mean ± standard deviation (mean ± SD). Statistical significance was denoted as p < 0.05 (*), p < 0.01 (**), and p < 0.001 (***).

## Results

### TRIM28 expression level in pancancer tissues compared with matched paracancerous tissues

**Figure 1:**
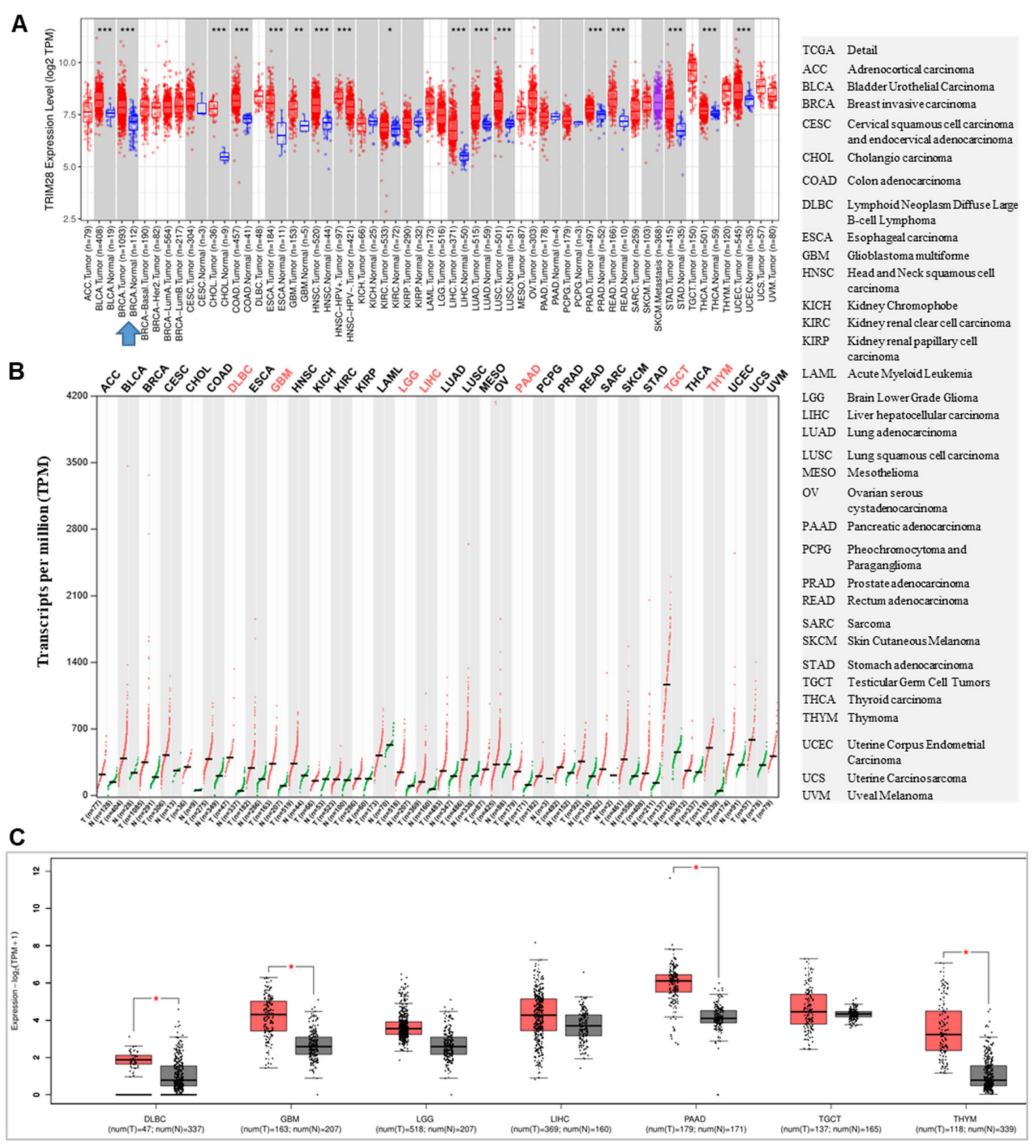
TRIM28 expression status in pan-cancer. A. The TRIM28 expression status in tumors and their matched normal tissues across different cancer types is shown via TIMER 2.0. B. The TRIM28 expression status is depicted in tumors and their matched normal tissues across various cancer types using GEPIA2 with TCGA and GTEx data. C. The TRIM28 expression status in tumors and their matched normal tissues across different types of cancers is illustrated, with red colors derived from panel B (shown by box plots). The right panel indicates the full names of different cancer types, with grey box plots representing normal tissue and red box plots representing cancer tissue. * p < 0.05; ** p <0.01; *** p <0.001.

The level of TRIM28 expressions between tumor tissues and matched normal tissues in different cancer types were examined via TIMER 2.0, and found that the levels of *TRIM28* are significantly increased in BRCA, BLCA, COAD, CHOL, ESCA, GBM, KIRC, HNSC, LIHC, LUSC, LUAD, READ, PRAD, STAD, THCA, and UCES, but no significantly decreased were found when compared with the corresponding normal tissues in different types of cancer (TCGA normal and GTEx data) (Figure 1A). When we analyzed tumors and matched normal tissues in different types of cancer using TCGA and GTEx data via GEPIA 2, we observed that the levels of *TRIM28* were upregulated in all cancer types. They were significantly upregulated in DLBC, GBM, LGG, LIHC, PAAD, TGCT, and THYM, but no significant decreases were found when compared with the corresponding normal tissues (Figure 1B&C).

### TRIM28 expression is significantly increased in BRCA in tumor tissues compared with the matched healthy tissues

**Figure 2:**
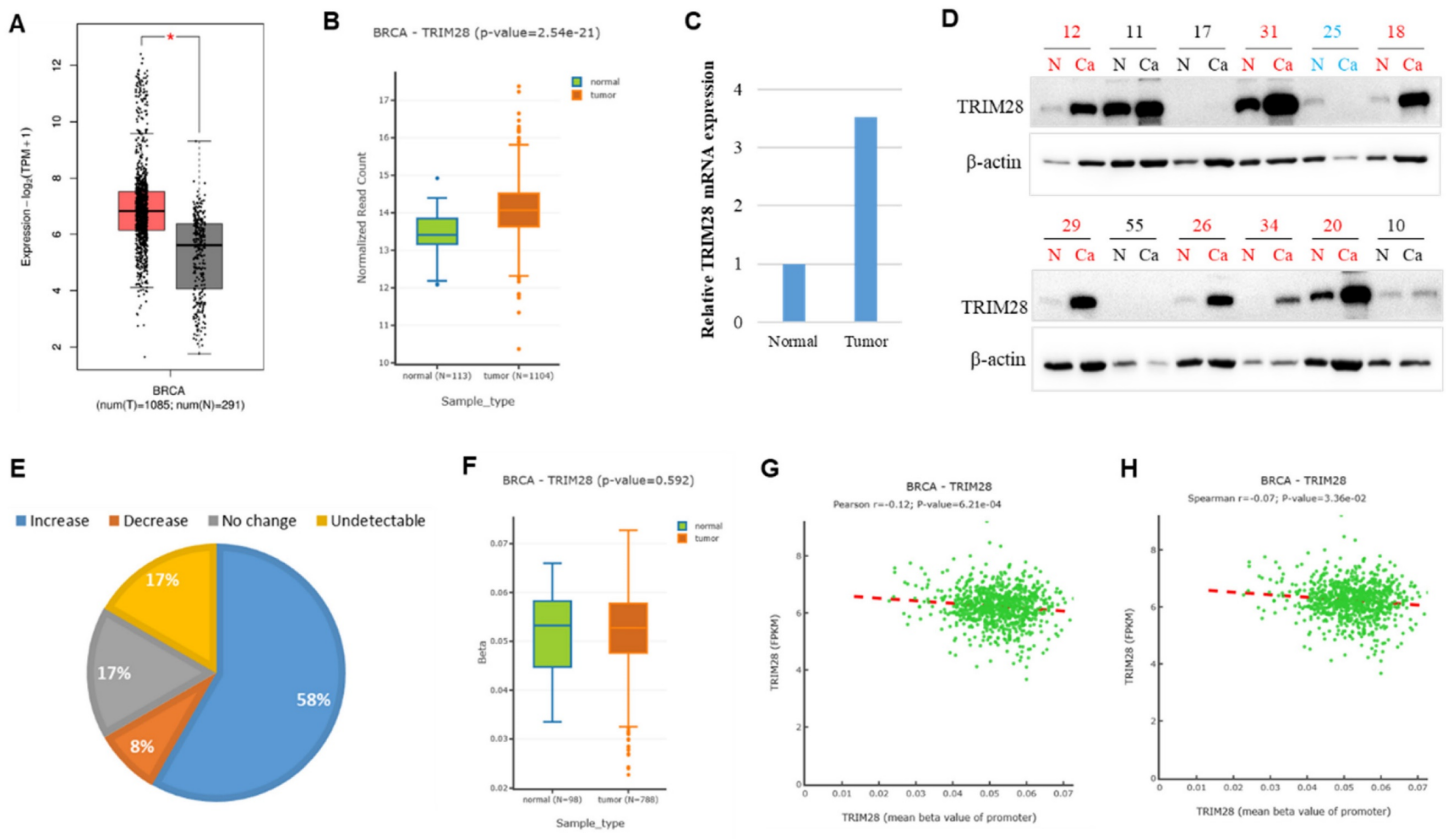
TRIM28 expression is increased in breast invasive carcinoma (BRCA) in tumor tissues compared with matched normal tissues. A. TRIM28 expression is increased in BRCA tumor tissues compared with the matched normal tissues via GEPIA 2 (shown by box plots). Grey box plots represent normal tissue, and red box plots represent cancer tissue (* p < 0.05). B. TRIM28 expression is increased in BRCA tumor tissues compared with matched normal tissues via DNMIVD: DNA Methylation Interactive Visualization Database (as illustrated by box plots). Green box plots represent normal tissues, and red box plots represent cancer tissues. C. Quantitative RT-PCR results in BRCA indicate differences in gene expression between tumor tissues and their corresponding normal tissues. D. Western blotting results from 12 BRCA samples in tumor tissues are compared with corresponding normal tissues. N: corresponding normal tissues, Ca: cancer tissues. Red color indicates an increase, while green color indicates a decrease in TRIM28 protein in tumor tissues compared with corresponding normal tissues. E. Quantitative Western blotting results in BRCA reveal differences in TRIM28 protein expression levels between tumor tissues and their corresponding normal tissues. F. TRIM28 methylation at the promoter region is depicted by boxplot in BRCA, comparing tumor tissues with corresponding normal tissues via DNMIVD. Green box plot: normal tissue, and red box plot: cancer tissue. Pearson (G) and Spearman (H) correlations between TRIM28 expression and methylation of the TRIM28 gene promoter are presented. FPKM means fragments per kilobase of exon per million fragments mapped.

Then, we carried out a further analysis of the TRIM28 level in BRCA by comparing tumor tissues with the corresponding healthy tissues via both GEPIA 2 and DNMIVD. Our findings revealed that TRIM28 expression is significantly upregulated in BRCA tumor tissues compared with the matched healthy tissues (Figure 2A&B). To further validate the bioinformatics results, we collected 33 samples and conducted quantitative RT-PCR performed in BRCA Chinese patients, analyzing both tumor tissues and the matched healthy tissues. The results showed that *TRIM28* mRNA expression is significantly increased by 3.5 folds in tumor tissues compared to the corresponding healthy tissues (Figure 2C). We also collected 12 BRCA samples of tumor tissues and the corresponding healthy tissues. Western blotting was carried out, and the results showed that TRIM28 protein expression is upregulated in 7 samples of tumor tissues (58%), downregulated in only 1 sample of tumor tissues (8%), showed no change in 2 samples of tumor tissues (17%), or was undetectable in 2 samples of tumor tissues (17%) when compared with the corresponding normal tissues, respectively (Figure 2D&E).

**Figure 3:**
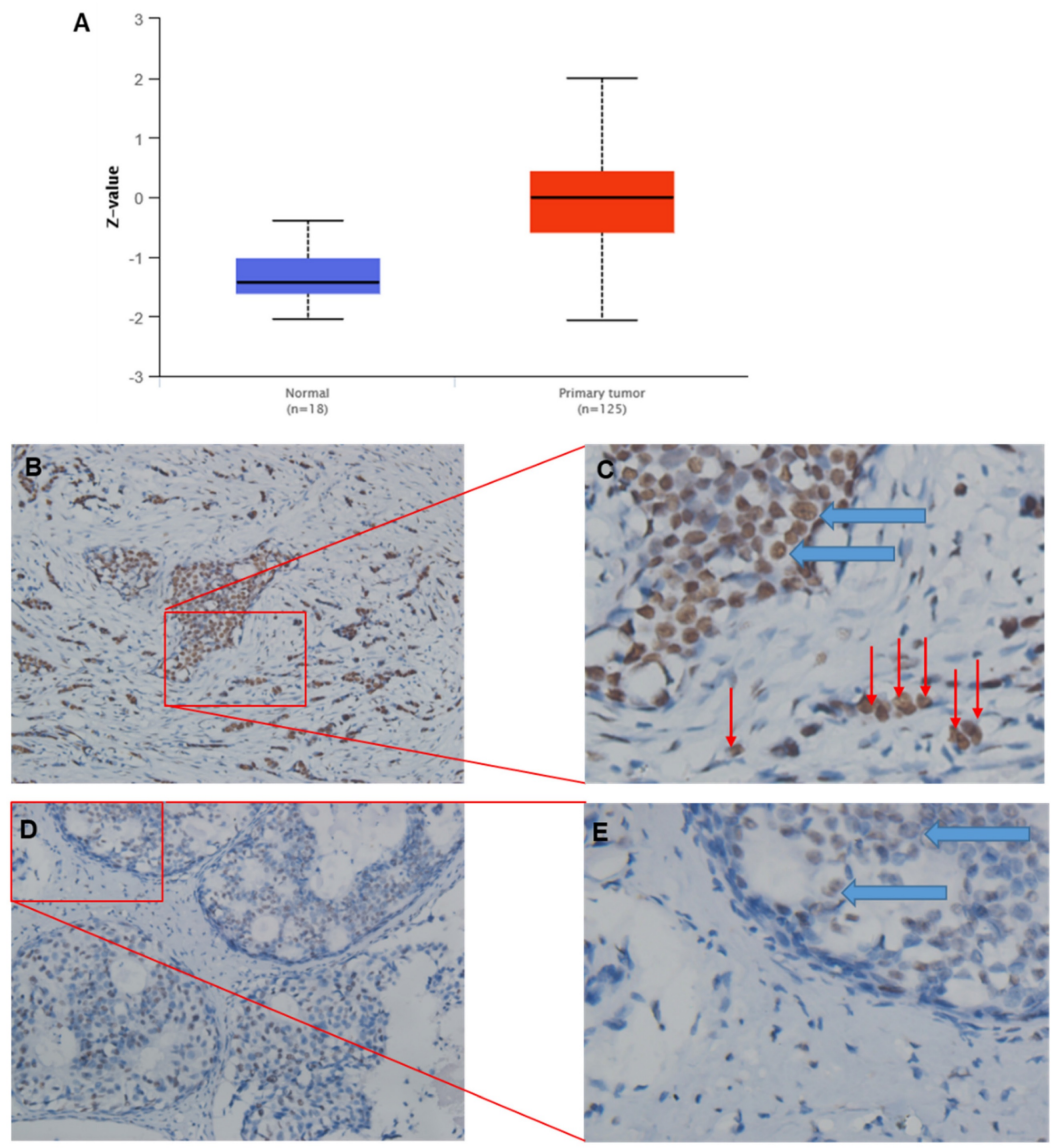
Representative images of TRIM28 protein expression by IHC (immunohistochemistry) in BRCA tumor tissues. A. TRIM28 protein expression is significantly upregulated in CPTAC BC tissues compared with corresponding healthy tissues. B. Representative images show a higher TRIM28 protein level in BRCA tumor tissues. D. Representative images depict a lower level of TRIM28 protein in BRCA tumor tissues. C&E. Enlarged images of panels B and D, respectively. Green arrows indicate breast ducts, while red arrows indicate invasive tumor cells.

For consistency, protein expression levels for TRIM28 are also significantly upregulated in CPTAC BC tissues compared with corresponding normal tissues (Figure 3A). IHC results might support the above conclusion, where green arrows indicate tumor cells inside the breast duct, while red arrows indicate invasive tumor cells (Figure 3B~E). In addition, TRIM28 is mainly localized in the nuclei (Figure 3B~E).

Differentially methylated genes could affect their expressions. To further analyze the possible regulation mechanism for TRIM28 expression, TRIM28 methylation at the promoter region was performed in BRCA tumor tissues and the matched healthy tissues via DNMIVD. The results showed that TRIM28 methylation in BRCA tumor tissues is slightly decreased but not significantly compared to the corresponding normal tissues (Figure 2F, p value=0.592). Both Pearson and Spearman correlation coefficients between methylation of TRIM28 and fragments per kilobase of exon per million fragments mapped (FPKM) were calculated. The results indicated that the expression and methylation of TRIM28 are reversely correlated (Figure 2G&H, p value<0.001).

### TRIM28 expressions in different pathological stages of BRCA

**Figure 4:**
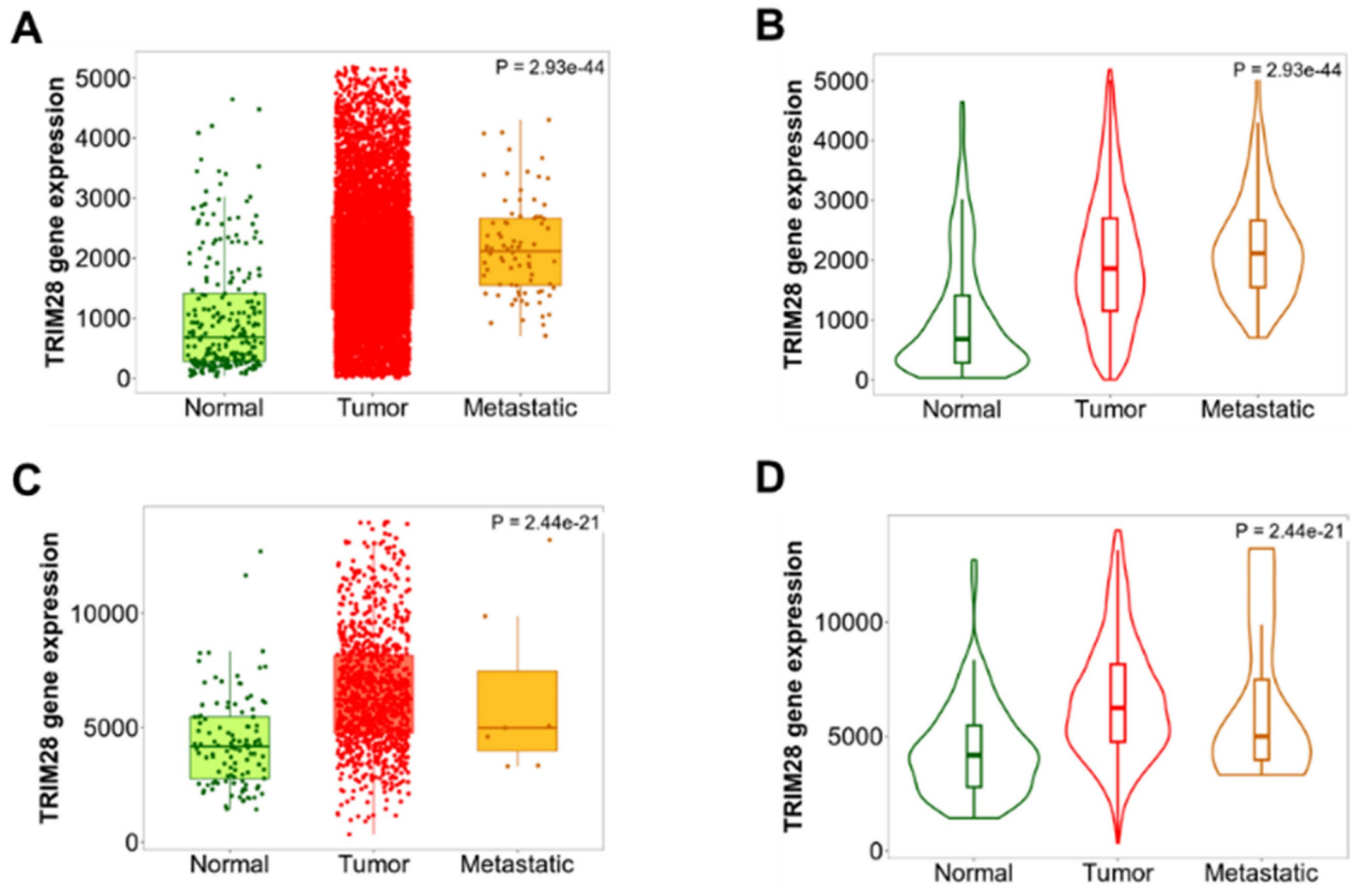
TRIM28 expression levels in different pathological stages in BRCA. A& B. Differences in TRIM28 levels among normal, tumor, and metastatic tissues have been demonstrated in BRCA, and analyzed using gene chip data. C&D. Differences in TRIM28 levels among normal, tumor, and metastatic tissues have been depicted in BRCA, and analyzed using RNA-Seq based data. Panel A&C: boxplots, B&D: violinplots.

We'd like to know whether TRIM28 is differentially expressed in different pathological stages in BRCA, TNM plotter was used to analyze differences in TRIM28 expression among tumor, metastatic, and normal tissues by gene chip data. The results are shown in Figure 4, where TRIM28 expression is remarkably associated with the pathological stages (Figure 4A~D), specifically, the TRIM28 expression level is significantly increased in metastatic BRCA (Figure 4A~B), indicating that TRIM28 expression might be correlated with metastatic / invasive progression.

### TRIM28 expression level is a prognostic marker for survival

**Figure 5:**
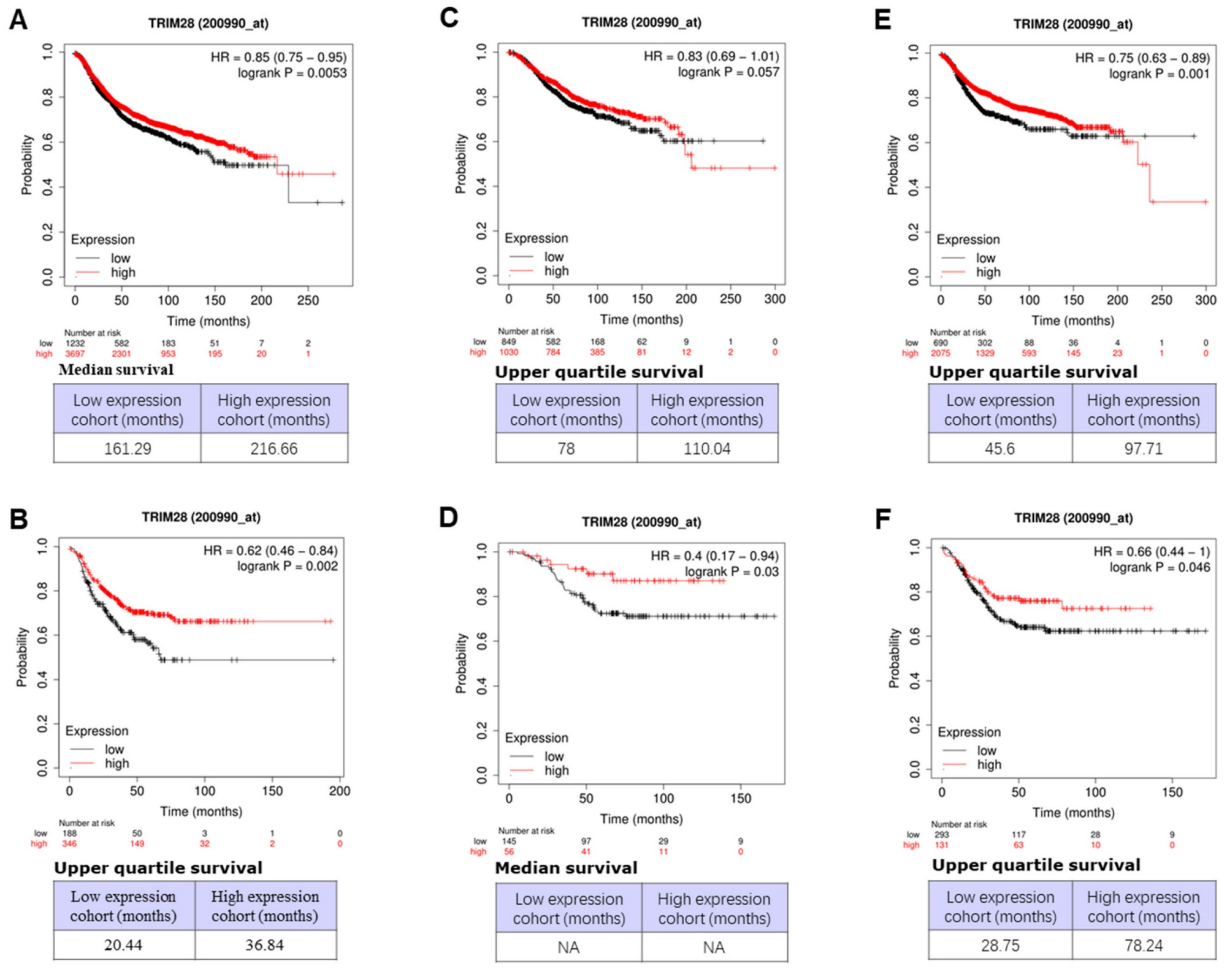
Association between TRIM28 expression level and OS, RFS, and DMFS in patients with BC. A. BC patients with RFS. B. TNBC patients with RFS. C. BC patients with OS. D. TNBC patients with OS. E. BC patients with DMFS. F. TNBC patients with DMFS. Kaplan-Meier survival curves of patients with high and low TRIM28 levels BCs and TNBC patients are shown.

We then investigated the associations between TRIM28 level and outcomes of survivals, including overall (OS), recurrence-free (RFS), and distant metastasis-free (DMFS), in different BCs or triple-negative breast cancer (TNBC) patients using an online database (Dataset Affy ID:200990_at) through Kaplan-Meier Plotter with the auto-select best cutoff. The results are shown in Figure 5, where a high level of TRIM28 is significantly associated with a long RFS of BC (Figure 5A, p<0.0053) and a long RFS of TNBC (Figure 5B, p<0.002), a long OS of TNBC (Figure 5D, p<0.003), a long DMFS of BC (Figure 5E, p<0.001) and a long DMFS of TNBC (Figure 5F, p<0.046), respectively. However, the high level of TRIM28 isn't significantly associated with a long OS of BC (Figure 5C, p<0.057).

**Figure 6:**
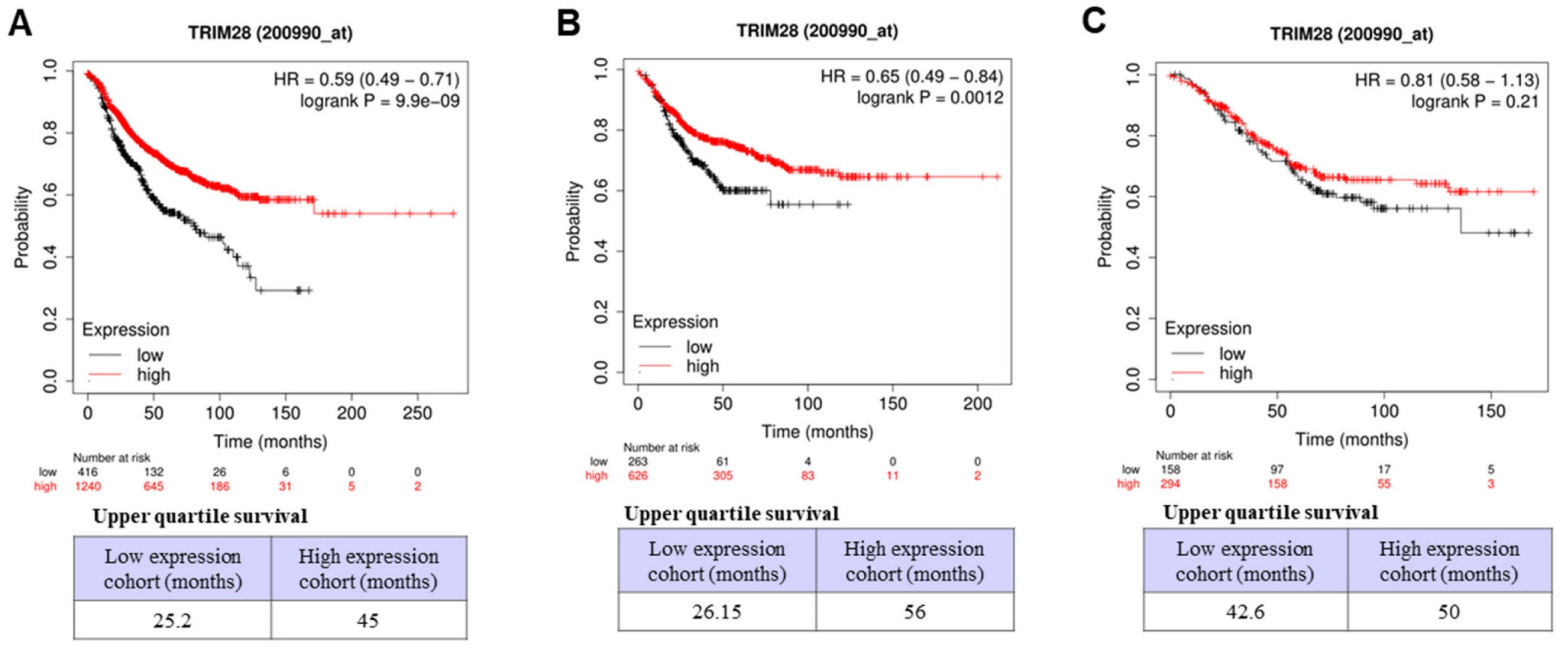
Association between TRIM28 expression level and RFS, OS, and DMFS in BC patients with lymph node-positive. A. BC patients with RFS with lymph node-positive. B. BC patients with DMFS with lymph node-positive. C. BC patients with OS with lymph node-positive.

We further investigated the associations between TRIM28 levels and outcomes of survivals of RFS, OS, and DMFS in BC with lymph node-positive status by the same database (Dataset Affy ID:200990_at) through Kaplan-Meier Plotter with the auto-select best cutoff. The results indicated that, in BC with lymph node-positive, a high level of TRIM28 is significantly associated with a long RFS (Figure 6A, p<0.001) and a long DMFS (Figure 6B, p<0.0012), but not with a long OS (Figure 6C, p=0.21). Therefore, TRIM28's high expression might be a prognostic marker for long survival in patients with TNBC or advanced / invasive breast carcinoma.

### CD inhibits breast tumor growth and progress *in vivo* likely through TRIM28

CD has effects on tumor progression including proliferation, migration, and invasion both *in vitro* and *in vivo* 26, 29. We aimed to establish a BC mouse model by injecting 4T1 cells, a highly invasive mouse BC cell line, subcutaneously into mice. The mice were treated with CD after a five-days injection, once the tumors reached approximately ~10mm^3^ (1-3 mm in detentions). The whole body weights of the mice were not dramatically affected by CD treatment. The sizes of the tumors in all groups increased during the experimental period.

**Figure 7:**
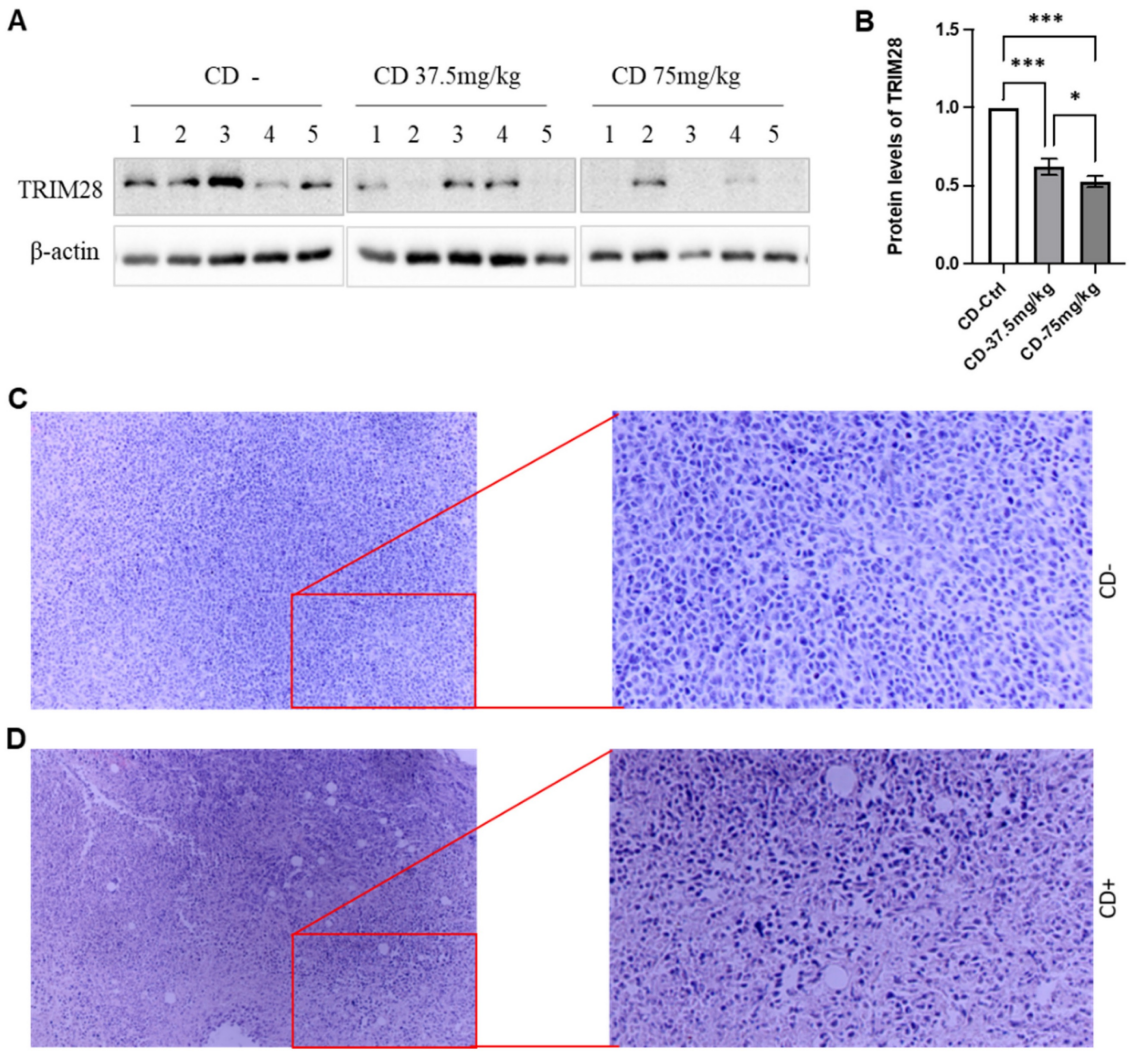
CD suppresses breast tumor growth in vivo likely through TRIM28 expression. A. In vivo, CD suppresses TRIM28 expression in breast tumors. B. Quantitative results for panel A. * p < 0.05; *** p <0.001. C. Representative H&E staining results for control group without CD treatment. D. Representative H&E staining results for CD treatment group. Right panels show enlarged images in Figure 7C&D.

At the endpoints, the animals were sacrificed, and the size and weight of the tumors were measured. CD treatment remarkably decreased both tumor size and weight, respectively, in a dose-dependent manner (Data not shown). We also extracted whole proteins from the tumor tissues and performed western blotting assays to monitor the effect of CD on TRIM28 expression. As shown in Figure 7A&B, the TRIM28 protein level is decreased by the treatment of CD in a dose-dependent manner among the treatments (Figure 7A&B). These findings were in accordance with the immunostaining results (Data not shown). Meanwhile, H&E staining indicated that CD treatment presented tumor cell necrosis and showed many empty bubbles (Figure 7C&D). Altogether, these findings imply that CD represses BC growth and metastasis, at least partially via suppressing TRIM28 expression.

## Discussion

In the current study, the TRIM28 expression level was systematically analyzed and found to be increased in all cancer types. It was significantly increased in BRCA, BLCA, COAD, CHOL, ESCA, GBM, KIRC, HNSC, LIHC, LUSC, LUAD, READ, PRAD, STAD, THCA, UCES, DLBC, LGG, PAAD, TGCT, and THYM by comprehensive analysis using both TIMER 2.0 and GEPIA 2 databases, in pancancerous tissues compared with the matched paracancerous tissues. Further analyzing the TRIM28 expression in BRCA, we revealed that TRIM28 expression is significantly increased in tumor tissues compared with the matched normal tissues. These bioinformatics results were further validated by RT-qPCR, IHC, and western blotting, demonstrating TRIM28's roles in the progression of pan-cancers, particularly in BRCA. Differentially methylated genes could affect their expressions, TRIM28 methylation at the promoter region was slightly decreased but not significantly compared with the matched normal tissues. Pearson and Spearman correlation results found that the expressions and methylation of TRIM28 are inversely correlated, indicating that TRIM28 methylation at the promoter region could affect TRIM28 expression levels. These results demonstrate that TRIM28 methylation may be one of the mechanisms for its upregulation, and further study might be needed to confirm this. Moreover, TRIM28 expression is significantly associated with pathological stages among tumor, metastatic, and normal tissues, and is significantly increased in metastatic BRCA in gene chip data, further supporting the role of TRIM28 in the metastatic / invasive progression for patients with BRCA.

However, when we analyzed the associations between TRIM28 level and outcomes of survivals in patients with different BCs, such as TNBC, BC with lymph node-positive via Kaplan-Meier Plotter, we found that a high level of TRIM28 is remarkably associated with a long RFS of BC (p<0.0053) and a long RFS of TNBC (p<0.002), a long OS of TNBC (p<0.003), a long DMFS of BC (p<0.001), and a long DMFS of TNBC (p<0.046), respectively. However, the high level of TRIM28 wasn't significantly associated with a long OS of BC (p<0.057). Again, in BC with lymph node-positive status, a high level of TRIM28 expression is significantly associated with a long RFS (p<0.001) and a long DMFS (p<0.0012), but not with a long OS (p=0.21). Unlike TRIM28 upregulation associated with poor prognosis of gastric cancer, HCC, or ovarian cancer 21-24, TRIM28's high expression might be a good prognostic marker for long survival in TNBC or advanced / invasive breast carcinoma (BRCA). Please note that we didn't find any significant correlation with survival outcomes in BC via GEPIA2. In addition, there is only one dataset (Affy ID:200990_at) available via Kaplan-Meier Plotter. Of course, it will be better if there are more data to support the aforementioned conclusion as a prognostic marker. And we plan to conduct more studies in the future. Nevertheless, the complex nature of TRIM28's contribution to different types of cancer makes TRIM28 an achievable candidate for targeting anti-cancer therapy 10, 11.

CD, an anti-cancer drug derived from a natural product of *Ophiocordyceps sinensis* (Berk.) or *Cordyceps militaris* Link, has been reported to exert effects on tumor progression, including proliferation, migration, invasion, both *in vitro* and *in vivo* 26-29. Our results found that TRIM28 is downregulated by CD treatment in a dose-dependent manner *in vivo*, demonstrating that CD represses BC growth and metastasis, at least partially, via suppressing TRIM28.

In conclusion, TRIM28 shows a high level in BRCA tissues and is correlated with metastatic / invasive progression. TRIM28 high expression would be a good prognostic marker for long survival in TNBC or advanced / invasive breast carcinoma (BRCA), but further study could be conducted. CD may inhibit TRIM28 expression and prevent BC progression. Thus, TRIM28 might be a potentially valuable molecular marker for predicting the progression, prognosis, and therapeutics of patients with BRCA.

## Figures and Tables

**Table 1:** Primer sequences for RT-qPCR used for Human tumor samples

Gene name	Primer name	Sequence (5'-3')	Probe
TRIM28	TRIM28L49	atggtgcagacagcactgg	49
	TRIM28R49	gcagtacacgctcacatttcc	
18S	Q18S-48L	gcaattattccccatgaacg	48
	Q18S-48R	gggacttaatcaacgcaagc	
